# Dentinitis

**Published:** 1867-04

**Authors:** Henry S. Chase


### DENTINITIS.

*(Inflammation of Dentine—Sensitive Dentine.)*

BY HENRY S. CHASE.

I have had many cases this winter of sensitive dentine at the neck of the tooth. Patients complain of return pain on touching, even lightly with a pin or the finger nail, that portion of the tooth immediately below the edge of the gum. This is the case even where there is no *apparent* corrosion or disintegration of tissue. Undoubtedly the microscope would reveal in these cases, a definite chemical disintegration of either the enamel, the dentine, or the cementum. In some cases it was so severe that cold water was as unbearable as the contact of hard substances. In some, the patients came with the request to have the teeth extracted. Fortunately I have had a good deal of experience with this class of cases, and commenced many years ago to treat them with creosote, and I do not remember of failing in a single instance by its application directly to the inflamed portion one, two or three times. With a little cotton on the point of an excavator and wet it with creosote, then rub it back and forth eight or ten times over the diseased part.

